# Exploring the ROS reduction strategies in chronic lupus management

**DOI:** 10.3389/fimmu.2024.1346656

**Published:** 2024-02-20

**Authors:** Kannika Parameshwari Kannan, Smiline Girija A.S.

**Affiliations:** Department of Microbiology, Saveetha Dental College and Hospitals, Saveetha Institute of Medical and Technical Sciences (SIMATS), Saveetha University, Chennai, Tamilnadu, India

**Keywords:** ROS, oxidative burst, SLE, lupus (SLE), radicals and free radical reactions

## 1 Introduction

Systemic lupus erythematosus (SLE) is an autoimmune disease affecting various systems by the generation of autoantibodies, particularly against nuclear antigens, and it is often considered a chronic lupus. SLE is a universal disease with an augmented risk of early death and, if left untreated, will cause irreversible damage to the affected tissue, causing complications in the affected individuals. Since the 1950s, the prevalence rate of SLE gradually increased from 63.2% to 91.4% until the early 2000s, with a survival rate of 10 years (1). SLE affects multiple organs and usually manifests at an increased rate in women than men, and the reported global incidence among the genders is in the ratio of 9:1 due to the preponderance of the female hormones playing a harmful role in SLE condition. SLE assaults the body’s own tissues, leading to extensive inflammation and tissue destruction in the affected organs such as blood vessels, brain, lungs, kidneys, joints, and skin. The mechanism of tissue damage is due to the development of antinuclear antibodies (ANAs) and thus is recognized as the “model” systemic autoimmune illness. The molecular interactions underlying SLE eventually cause the immune system to lose its ability to tolerate the nuclear auto-antigens. Since the exact cause of SLE is unclear, it is assumed that a combination of exogenous factors like smoking, viral infections, UV irradiation, and certain medications, along with genetic predisposition, causes the innate and adaptive immune systems to become dysregulated, resulting in SLE (2).

## 2 ROS mechanisms underlying SLE

### 2.1 Oxidative metabolism in SLE

**Figure 1:**
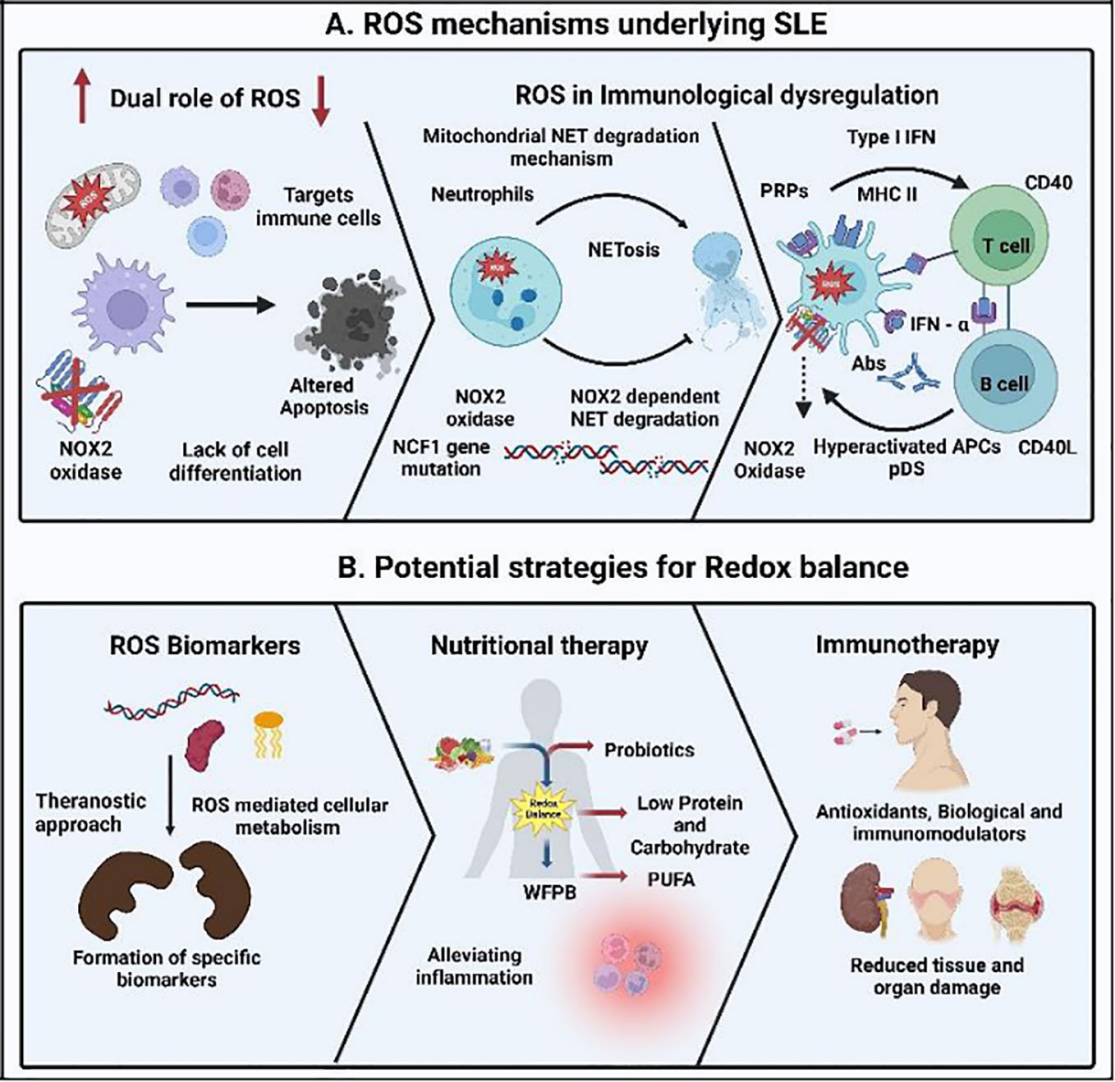
Reactive oxygen species (ROS) mechanisms underlying the immunology of the autoimmune disorder systemic lupus erythematosus (SLE) and its treatment strategy. **(A)** Increase and decrease in the ROS production leading to delay in the clearance of apoptotic material and enhancement of the cross-presentation of apoptotic cell-associated antigens leading to SLE. Lack of NOX2 activity inhibits the development of neutrophil extracellular traps (NETs) and the NET serine proteases, resulting in the destruction of pro-inflammatory mediators mediated through NCF1 mutations. Degradation of mitochondrial NETs influencing type I interferon (IFN) production under the control of mitochondrial ROS and activating the pattern recognition receptor (PRRs). This leads to the further signaling of plasmacytoid dendritic cells (pDCs) and increases the inflammatory cytokine release, resulting in auto-antibody formation in SLE, aiding tissue damage and multi-organ involvement. **(B)** Detection of specific ROS biomarkers, nutritional therapy, and immunotherapy in maintaining the redox balance will enhance the diagnosis, treatment, and health of SLE patients, respectively.

Numerous mechanisms have been proposed for the cell death underlying tissue damage and immunological dysregulation in SLE (**Figure 1**), but more research works are anticipated for a better understanding. It is a known fact that molecular oxygen is the primary force behind oxidative phosphorylation (OXPHOS), and the concept of the “molecular oxygen paradox” is inextricably linked to the production of extremely reactive and deadly by-products, known as reactive oxygen species (ROS) (3). Production of excess ROS or oxidants, termed oxidative stress, is often known to influence the antioxidant response of any cell, especially in SLE. ROS normally include non-radical oxidants like hydrogen peroxide (H_2_O_2_) and singlet oxygen (1O_2_) as well as oxygen free radicals such as superoxide anion radical (O_2_−) and hydroxyl radical (·OH). The primary endogenous enzymatic source of O_2_·− and H_2_O_2_ are through the electron transport chain (ETC) in mitochondria and transmembrane NADPH oxidases (NOXs) (4).

In SLE, ROS play an important role in cellular signaling and are considered hazardous reactive particles to the cells because they damage the intracellular proteins, lipids, and nucleic acids, producing toxic base pairs, leading to DNA damage. One such known DNA lesion is the 8-oxo-deoxyguanosine formed due to the transversion mutagenic lesion (5, 6). ROS also target the proteins through the oxidation of the amino acid, prosthetic groups, creation of cross-linkage, protein aggregates, and proteolysis. This may further lead to the inactivation of important proteins, arresting the critical metabolic processes. In the same way, vulnerable radical damage of the polyunsaturated fatty acids produces toxic alkyl radicals and hydro-peroxidized lipids. Further lipid peroxidations in the membrane phospholipids lead to damage of the cellular membrane (7, 8).

Clinical manifestations of ROS-induced SLE include fever, malaise, arthralgias, myalgias, headaches, and changes in appetite and weight. Additional systemic SLE illnesses included joint disorders (38%), oral ulcers (23%), central nervous system (CNS) issues (23%), and renal problems (38%). Raynaud’s pleuritis and sicca were more common in adult-onset SLE, whereas malar rash, renal involvement, ulcers/mucocutaneous involvement, proteinuria, seizures, urinary cellular casts, thrombocytopenia, hemolytic anemia, fever, and lymphadenopathy were more common in childhood-onset SLE (9).

### 2.2 NADPH oxidase enzymes and respiratory burst in SLE

It is a known fact that the ROS are crucial for several biological functions such as immunity, signaling, and metabolism. Such cellular ROS typically arise from superoxide, through two vital sources in a cell: 1) through the oxidative phosphorylation in mitochondria and 2) NOX enzymes. During phagocytosis and normal cellular functions, superoxide is generated by NADPH oxidase enzymes and is a part of the respiratory burst. Additionally, NOX-derived ROS control multiple immunological functions, including cell signaling, type I interferon modulation, antigen presentation, antigen processing, pathogen clearance, phagocytosis, and inflammasome regulation (10). Also, during the deficit of ROS, various malfunctions may lead to the progression of SLE.

In addition to NOX2 deficiency linked to impaired pathogen antigen clearance, it is also linked to modified T-cell responses in SLE. Furthermore, the NOX2 complex-induced SLE is the common co-occurrence of cutaneous lupus erythematosus, a hereditary disease resulting from reduced ROS generation from the NOX2 complex. It is evidenced in mouse models of SLE that the NOX2 complex can act as a negative regulator of autoantibody synthesis and autoimmune inflammation. The same mechanism is also documented in the pristine induced lupus in mice with a dysfunctional NOX2 complex causing the secondarily necrotic cell (SNEC)-derived material to be bypassed from regulatory to inflammatory phagocyte subsets. This suggests that NOX2-dependent inflammatory re-routing plays a significant role in the development and maintenance of exacerbated SLE in people whose NOX2 complex activity is low (11). However, the actual mechanism is not entirely elucidated.

### 2.3 Malfunction of NCF1 and NOX2 in SLE

Patients with SLE lack phagocytic oxidative burst because they have mutations in essential phagocyte NOX2 complex subunits. Neutrophil cytosolic factor 1 (NCF1) is a significant genetic factor linked to autoimmune disorders, and NOX2-derived ROS are observed specifically in the mutated NCF1 gene, contributing to the malfunctioning of NCF1 in lupus. It also indicates the protective role of plasmacytoid dendritic cell (pDC)-derived ROS in SLE progression when NCF1-dependent ROS shortage is observed. In the human genome, complex 7q11.23 region contains NCF1, which codes for the p47phox/Ncf1 protein of the (NADPH) oxidase (NOX2) complex, a necessary component for the induction of ROS (12). Autoimmune disorders like SLE are significantly linked to NCF1 mutations that result in decreased generation of ROS as well. In this context, a single-nucleotide polymorphism (SNP) has been found in NCF1 gene, coding for the NCF1 protein, also referred to as p47phox and a crucial NOX2 subunit (denoted NCF1-339). In the form of both odds ratio and allelic frequency, the most significant genetic relationship for SLE is represented by the defective NCF1-339 allele, which results in reduced NOX2 complex function (13).

### 2.4 Dual role of ROS in SLE

Chronic inflammation in SLE can result in an overabundance of ROS, triggering oxidative stress and leading to DNA damage with localized endothelial and epithelial cell necrosis. This can further trigger the release of self-reactive T and B cells, accelerating the inflammatory response (14). Moreover, increased apoptosis and the potential for delaying the removal of apoptotic cells are linked to ROS generation (15). Studies in animals document the overproduction of ROS in SLE (16) and increased oxygen intermediates in lupus mouse macrophages (17). These mice exhibited increased formation of ROS in their tissues, pulmonary hemorrhages, elevated glomerulonephritis, and increased levels of antibodies against dsDNA, histones, and Sm/RNP. The model suggests that abnormal phagocytosis or incapacity to create NETs could be potent causes of the disease progression. Lupus-prone NZBWF1 animals have increased NADPH oxidase subunit expression in aortic tissues, increased NADPH oxidase activity in aortic rings, and higher systolic blood pressure (18).

Contrastingly, the lowering of ROS in evidenced studies has documented the harmful consequences of low ROS production in lupus animal models. Researchers Campbell and colleagues demonstrated that lupus was worsened in NOX2-deficient mice (5). Mice with the Ncf1m1j mutation and low ROS production spontaneously developed elevated levels of lupus-associated autoantibodies, significant IgG and complement C3 deposits in the glomeruli, and high expression of inflammation-related genes and IFN-stimulated genes (ISGs) (19, 20).

## 3 ROS role in altered apoptosis and as specific biomarkers in SLE

ROS generally appear in pathological conditions when scavenged prematurely, and ROS-mediated oxidative damage in SLE results in defective apoptotic control or programmed cell death. This may delay the apoptotic cell clearance, prolonging the synergic effect of ROS and apoptosis in the cellular macromolecules. ROS also aid in the production of neo-epitopes, inducing a broad spectrum of autoantibody formation and tissue damage in SLE. ROS primarily target the lipids in the cell membrane, initiating lipid peroxidation (LPO), leading to damage of the cell structure and function. This is evidenced in patients with SLE by the detection of elevated levels of malondialdehyde (MDA), a by-product of lipid peroxidation, in their blood, plasma, erythrocytes, and lymphocytes. Such an increase in MDA-modified proteins, anti-SOD, and anti-catalase antibodies in SLE patients induces oxidative stress with the onset of the disease (21).

Interestingly, these ROS indicators of oxidative damage can be considered for the development of biomarkers (22). MDA and 4-hydroxynonenal (4-HNE) are reported as diagnostic markers of lipid peroxidation, 8-hydroxy-2′-deoxyguanosine (8-OHdG) as a measure of oxidative DNA damage, and protein carbonyl groups as markers for protein oxidation (23, 24). The identification of such biomarkers for oxidative stress has sparked interest, but the complexity of the disease makes it challenging to select a single specific biomarker to capture oxidative damage and pathophysiological illness. However, in terms of anti-oxidative therapy, these oxidative stress indicators may be useful stress indicators in the near future to treat SLE.

## 4 Armory of immune cells dysregulating SLE

### 4.1 Functional influence of T cells and B cells in SLE

T cells play a major role in the initiation of SLE, and uncontrollably activated B cells are produced by T-cell dysregulation, which impacts peripheral immune tolerance. A similar state is observed in rheumatoid arthritis (RA) patients, where T cell-mediated dysregulation has become an important model system for studying the functional influence of ROS on T-cell functions. Notably, in RA, T cells undergo “reductive stress” and produce less ROS than healthy T cells (25). However, in SLE, T lymphocytes experience this ROS-lowering process, while the complete mechanism is not experimentally evidenced. In addition, some immune-metabolic pathways are initiated by OXPHOS, glycolysis, and the essential function of mitochondrial-derived reactive oxygen species (mtROS) as signals to regulate auto-reactive T-cell activation and differentiation. Therefore, auto-reactive T-cell function and pro-inflammatory responses may be inhibited by reprogramming T-cell metabolism by focusing on mtROS. During this process, T cells from SLE patients demonstrate mitochondrial dysfunction, as defined by mitochondrial hyperpolarization. It is a known fact that the T cells can only identify foreign and self-fragmented antigens in combination with MHC proteins. However, in SLE, T/B-cell activation, proliferation, and interactions are regulated by adaptor molecules, kinases, and cytokines, which are actively induced by abnormal T/B-cell signaling and are linked to the HLA-DR2 and HLA-DR3 alleles, leading to the development of autoantibodies and SLE susceptibility (26).

### 4.2 NETosis in SLE

The creation of neutrophil extracellular traps (NETs) is another tool in the armory of neutrophils, in addition to the traditional weaponry of phagocytosis and degranulation. NETs are produced by a distinct cell death mechanism known as “NETosis”. They consist of granular proteins, histones, and a few cytoplasmic proteins together with de-condensed chromatin DNA. NET generation was originally characterized as an active process that was highly structured and relied on NOX2 as a defense mechanism against the disease. Additionally, research has proven that mitochondrial ROS aid in the development of NETs (27). Although the exact molecular mechanisms for NET formation are yet unknown, the production of ROS by NADPH oxidase is elucidated. Research has demonstrated that the autoantigens LL37 and HMGB1, which are generated during the development of NETs, are crucial for both immunity and inflammation specifically in SLE. Together with DNA, they function as autoantigens and through Toll-like receptor 9 (TLR9), LL37–DNA complexes produced from NETs, it can directly activate the polyclonal B cells, which can boost the production of antibodies (Abs) (28). When compared to healthy individuals, the ability of SLE patient’s neutrophils to create NETs is higher. Numerous NET proteins directly contribute to tissue damage and correlate with SLE disease activity. SLE NET-encoded MMP9 stimulates endothelial MMP2, resulting in endothelial dysfunction and mortality. As a result, lupus nephritis develops in SLE patients as a result of cellular changes that promote vascular leakage *via* the intercellular junction protein such as VE-cadherin. Mesenchymal cells are also formed as a result of catenin signaling from elastase, which is related to the presence of a significant number of NETs in SLE pathogenesis (29).

### 4.3 Role of IFN-α in promoting SLE

IFNs function as an immunological adjuvant for a variety of immune responses such as promoting the generation of autoantibodies and pro-inflammatory cytokines by T cells, B cells, and monocytes. NETs can strongly stimulate pDCs to generate large amounts of IFN-α, which primes neutrophils for further NETosis. This loop facilitates the development and progression of SLE by interacting with other effects of IFN-α. However, the primary structural components of NETs are nuclear materials, such as histones and DNA. It makes sense that cytokines, such as IFN-α, can stimulate adult neutrophils *in vitro* and enable the development of NETs. SLE NETs stimulate pDCs to produce large levels of IFN- in a DNA- and TLR9-dependent manner. IFN-α may stimulate monocytes to become dendritic cells (DCs), and DCs identify antigens and continually generate IFN-α, which then spreads and promotes the autoimmune reaction in SLE (30).

In reality, ubiquitination reduces NETs from SLE patient subjects, and this causes more severe oxidative damage in NETosis (31). Reduction in ROS also leads to an increase in the JAK–STAT cascade, which is located downstream of the IFN receptor, triggering the expression of ISGs. Reduction in the formation of ROS is the most prominent single-nucleotide mutation linked to SLE. A deficiency of NOX2-derived ROS might cause delayed clearance of apoptotic material and enhanced cross-presentation of antigens associated with apoptotic cells. Insufficient NET synthesis also activates IFN production. To date, no well-defined mechanism prevails to explain why NOX2-derived ROS are absent.

## 5 Redox balance: a potential treatment strategy for SLE

Maintaining the redox balance in SLE-affected tissues is considered the potential way for SLE-affected patients. The harmful effect of ROS-induced tissue damage can be best alleviated by the following strategies.

### 5.1 Drugs to assist antioxidant homeostasis

Drugs assisting the antioxidant systems may aid in balancing the effects of ROS. Myeloperoxidase (MPO), glutathione peroxidase (GPx), catalase (CAT), and superoxide dismutase (SOD) are the most documented antioxidants. These enzymes are known to reduce the severity of radical toxicity and aid in balancing oxidative stress together with the existing exogenous and non-enzymatic antioxidants (32). However, the effectiveness of the available exogenous antioxidants in the form of natural or synthetic chemicals has not yet been fully evidenced.

### 5.2 NAC therapy

It has been demonstrated that NAC helps patients with SLE by suppressing mTOR (33). It has been demonstrated that mitochondrial ROS activate the mTORC1 complex. Notably, in a mouse model of lupus, mTORC1 and mitochondrial ROS are known to activate NLRP3 inflammasomes (34). Based on a study conducted by Suwannaroj et al. (2001), NAC-treated SLE mice had considerably fewer anti-DNA antibodies after 24 weeks than control mice and showed a slight improvement in mortality (35).

### 5.3 Nutritional therapy

Lifestyle-related factors play a major role in SLE, and numerous clinical and preclinical research studies have examined the effects of nutrition and diet on the inflammatory reaction and disease progression in SLE over the past 20 years as a topic of research. In SLE, nutritional therapy, which involves limiting the protein and carbohydrate intake as well as using nutritional supplements (such as vitamins, minerals, and polyphenols), is often considered a potential approach to managing inflammatory reactions (36). Whole-food, plant-based (WFPB) diets may be incorporated routinely for SLE patients so that there may be a reduction in the pro-inflammatory substances, hence ameliorating SLE symptoms. Nutritional therapy helps in lowering the co-morbidities and in raising the quality of life for SLE patients, with more preventive effects and fewer adverse effects (37). Additionally, probiotics and nutrition are particularly new approaches to preserving mitophagy and redox balance in SLE patients (38). This is also evidenced in animal models where the reduced calorie intake had prevented the disease development and anti-inflammation. Higher levels of polyunsaturated fatty acids (PUFAs) in the diet lower the risk of pregnancy and associated symptoms in women with SLE syndrome (39). Following scientific standardization, the administration of exogenous chemicals through food derivatives or dietary supplements was shown to be efficacious.

### 5.4 Application of immunomodulators

Reducing the severity and maintenance of tissue homeostasis is the major goal in the treatment of SLE patients using various biological agents, immunosuppressants, glucocorticoids, etc. Research on the therapeutic effects of biological medications is yet unclear, and the treatment of lupus has been facilitated by a deeper and more extensive knowledge of disease etiology. Several biological medicines targeting diverse molecular pathways have been put into therapy regimens for SLE patients who show refractoriness or intolerance to standard-of-care treatment. The goal of using biologicals in SLE treatment is to achieve illness remission and to create self-tolerance. The discovery of drugs that target particular pathogenic pathways should be further evidenced by experimental studies for a better knowledge of the disease heterogeneity and molecular mechanisms underlying the pathogenesis of SLE (40).

Proper first-line treatment should also be recommended for these patients, involving immunosuppressive medications such as mycophenolate mofetil (MMF), azathioprine (AZA), methotrexate (MTX), and cyclophosphamide (CYC). Notably, approved drugs for SLE are voclosporin and anifrolumab which are highly recommended for the patients. A chimeric antibody called rituximab (RTX) targets CD20, which is expressed in B cells and is also effective in decreasing B-cell activation. Even though two RTX phase II/III trials in SLE failed to reach the primary objective, the adverse effects of such immunomodulation need to be further experimentally elucidated (41).

Personalized medicine needs to be tailored to the treatment decisions based on the genetic background of SLE patients. Modern lupus care approach is based mainly on treating SLE rapidly and preventing damage while reducing the usage of strong medicines such as immunomodulators. Recommendations on the application of the monoclonal antibodies by the National Institute for Health and Care Excellence (NICE) may be also advised for SLE. More evidence-based research studies are the need of the hour to introduce novel drugs for SLE, as no new drugs for the treatment of disease have been introduced in the pharmaceutical industry for more than 12 years (42).

## 6 Conclusion

In conclusion, developing an efficient treatment strategy for SLE patients is extremely challenging due to the ROS-based disease complexity and heterogeneity. Immunomodulators and precision-based therapy based on transcriptome analysis for detecting immune responses and gene signatures may be beneficial for SLE patients. An in-depth knowledge of the biology of SLE in association with ROS is the need of the hour to lay the foundation for the identification of specific novel biomarkers for SLE to grade the disease level and theranostic approach.

## Author contributions

KK: Conceptualization, Data curation, Formal analysis, Investigation, Methodology, Writing – original draft. SG: Conceptualization, Data curation, Formal analysis, Funding acquisition, Investigation, Methodology, Project administration, Resources, Software, Supervision, Validation, Visualization, Writing – original draft, Writing – review & editing.
